# Isolation of new Brazilian giant viruses from environmental samples using a panel of protozoa

**DOI:** 10.3389/fmicb.2015.01086

**Published:** 2015-10-06

**Authors:** Fábio P. Dornas, Jacques Y. B. Khalil, Isabelle Pagnier, Didier Raoult, Jônatas Abrahão, Bernard La Scola

**Affiliations:** ^1^Unité de Recherche sur les Maladies Infectieuses et Tropicales Emergentes CNRS 7278 UMR 6236 – IRD 3R198, Faculté de Médecine, Aix-Marseille UniversiteMarseille, France; ^2^Laboratorio de Vírus, Departamento de Microbiologia, Instituto de Ciências Biológicas, Universidade Federal de Minas GeraisBelo Horizonte, Brazil

**Keywords:** Mimivirus, Marseillevirus, Pandoravirus, Megavirales, giant virus, isolation, acanthamoeba

## Abstract

The Megavirales are a newly described order capable of infecting different types of eukaryotic hosts. For the most part, the natural host is unknown. Several methods have been used to detect these viruses, with large discrepancies between molecular methods and co-cultures. To isolate giant viruses, we propose the use of different species of amoeba as a cellular support. The aim of this work was to isolate new Brazilian giant viruses by comparing the protozoa *Acanthamoeba castellanii*, *A. polyphaga*, *A. griffini*, and *Vermamoeba vermiformis (VV)* as a platform for cellular isolation using environmental samples. One hundred samples were collected from 3 different areas in September 2014 in the Pampulha lagoon of Belo Horizonte city, Minas Gerais, Brazil. PCR was used to identify the isolated viruses, along with hemacolor staining, labelling fluorescence and electron microscopy. A total of 69 viruses were isolated. The highest ratio of isolation was found in *A. polyphaga* (46.38%) and the lowest in *VV* (0%). Mimiviruses were the most frequently isolated. One Marseillevirus and one Pandoravirus were also isolated. With Brazilian environmental samples, we demonstrated the high rate of lineage A mimiviruses. This work demonstrates how these viruses survive and circulate in nature as well the differences between protozoa as a platform for cellular isolation.

## Introduction

The viruses from the proposed order Megavirales have been described infecting eukaryotic hosts from different taxa (Colson et al., 2012). Although giant viruses of phagocytic protists may infect a wide variety of hosts, each species apparently has host specificity (Colson et al., 2012). As they have been isolated in a limited number of protozoa used to support co-culture, the natural host is usually unknown for most of them (Colson et al., 2012). Members of the two recently created families Mimiviridae and Marseilleviridae have been detected and isolated from highly diverse environments including human samples (La Scola et al., 2003, 2008; Boyer et al., 2009; Arslan et al., 2011; Boughalmi et al., 2013a,b,c; Ngounga et al., 2013; Saadi et al., 2013a,b; Campos et al., 2014; Dornas et al., 2014a; Scheid et al., 2014; Andrade et al., 2015; Assis et al., 2015; Reteno et al., 2015), and to date, considering protist hosts, these viruses multiply only in *Acanthamoeba* sp. (La Scola et al., 2003; Raoult et al., 2004; Colson et al., 2013). More recently, a new Megavirales member, the asfarviridae-related Faustovirus, was isolated using *Vermamoeba vermiformis (VV)* to support a culture showing possible high diversity between protists and giant viruses (Reteno et al., 2015).

The discovery of these groups of giant viruses has been delayed, since prior to the last decade, conventional techniques for viral isolation began with filtration to inoculate small viral particles only and thus missed viruses with a size comparable to bacteria (La Scola et al., 2003). Initially, concentration by filtration followed by direct inoculation was proposed (La Scola et al., 2008). Antibiotics in amoeba co-culturing procedures were subsequently added, with the goal of reducing bacterial contamination (La Scola et al., 2010). Other methods and modifications have also been reported (Arslan et al., 2011; Dornas et al., 2014b). Currently, to efficiently isolate giant viruses, new methods combining molecular biology techniques with high throughput strategies have been designed (Boughalmi et al., 2013a; Pagnier et al., 2013).

Most groups have used *Acanthamoeba castellanii (AC)* and *Acanthamoeba polyphaga (AP)* as cellular supports to isolate new giant viruses (La Scola et al., 2003; Philippe et al., 2013; Campos et al., 2014; La Scola, 2014; Scheid, 2014; Scheid et al., 2014), but to date no comparison between different amoebal supports have been reported. In addition, in Brazilian samples only lineage A mimiviruses have been isolated to date (Campos et al., 2014; Andrade et al., 2015; Assis et al., 2015). The aim of this work was therefore to compare different co-cultures with AC, *AP*, *Acanthamoeba griffinii (AG)*, and *VV* as cellular supports to search for new lineages of *Mimiviridae* and other giant viruses in Brazilian environmental samples.

## Materials and Methods

### Samples

**FIGURE 1:**
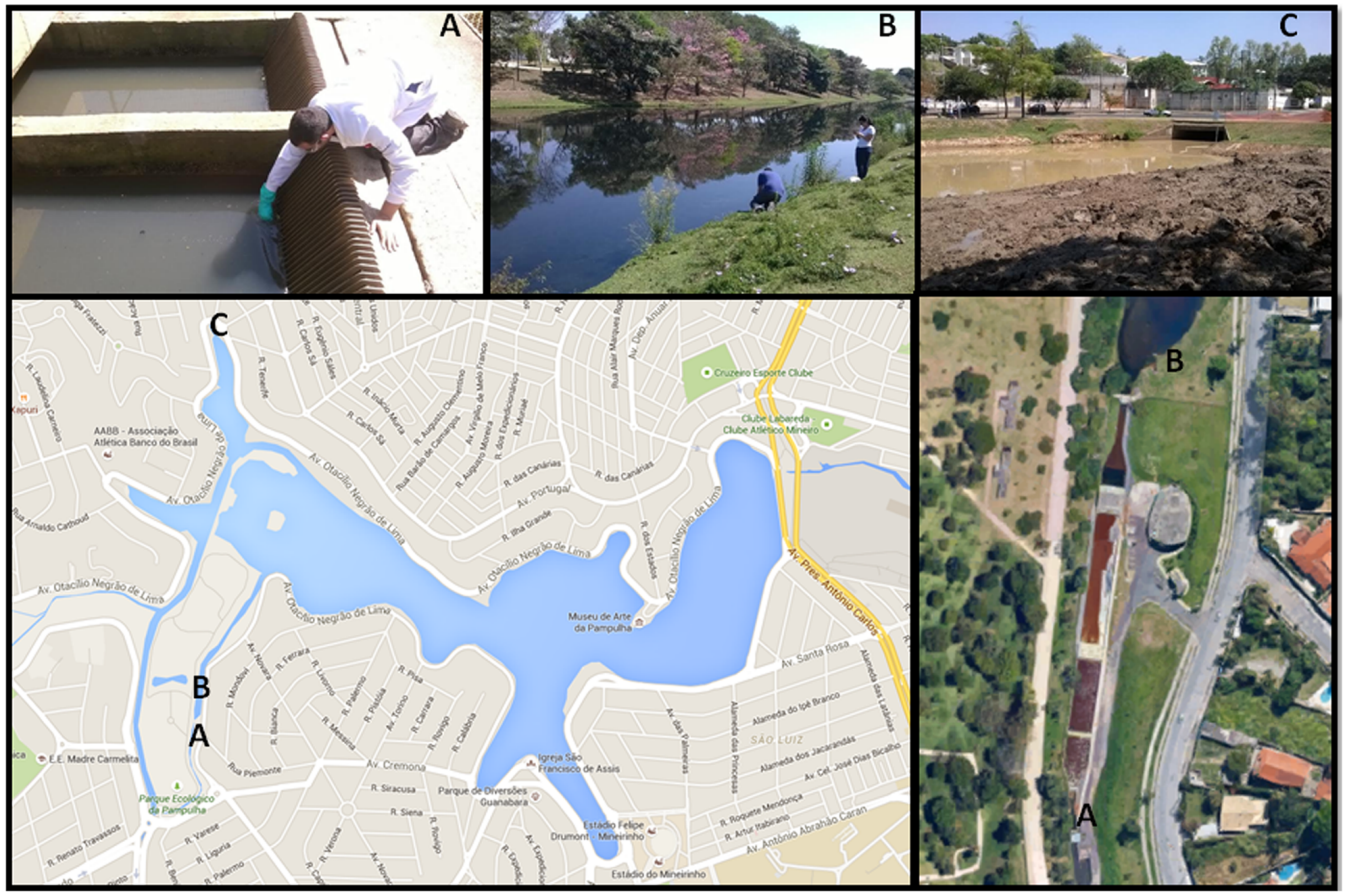
**Pictures from the three areas where the environmental samples were collected represented by letters (A–C). (A)** Point 1, before the chemical treatment; **(B)** Point 2, after the chemical treatment; **(C)** Point 3, 3 is an isolated area of the lagoon (Google Earth).

In September 2014, one hundred samples, including sewage, sludge, water, wet soil, and lake sediment, were collected in sterile tubes from three different areas of the Pampulha lagoon in Belo Horizonte city, Minas Gerais state, Brazil. The samples were numbered one to 100 and stored at 4°C until the inoculation procedures. Area 1 is the sewage treatment station and the samples were collected before chemical treatment of the sewage. In Area 2, the samples were taken where water is received after chemical treatment. The third area (Area 3) was chosen because of its distant location from the sewage treatment station. Area 3 is an isolated area of the lagoon, which is rich in organic matter and receives only rainwater. In this area the soil was on the edge of the lake, removed from the deepest part of the lake (**Figure 1**).

### Samples Treatment

Initially, the samples were divided in two groups, one with sediment-free water and the other with a high concentration of sediment and soil. Samples with only water and no sediment were inoculated directly in co-cultures. The samples with sediment and soil were pretreated by adding four times their volume of sterile Page’s Amoeba Saline (PAS), vortexing them, and allowing them to settle before decanting and later filtering via a paper filter to remove large particles of sediment. The samples were stored at 4°C until the inoculation procedures.

### Culture Procedures

The amoeba supports for co-culture were *AC* (strain NEFF), *AP* (strain LINC AP1), *Acanthamoeba griffini (AG;* strain ATCC 50702), and *VV* (strain CDC 19). The amoeba strains were kept in a 75 cm^2^ cell culture flask with 30 ml of peptone-yeast extract-glucose medium (PYG) at different temperatures according to species specificity. The temperatures were 28°C for *AC* and *VV* and 30°C for *AP*. After inoculation, *AP* and *AC* were kept at 32°C and *VV* at 30°C, respectively. *AG* was also kept at 35°C before and after inoculation. After 24 h of growth, cells were harvested and pelleted by centrifugation. The supernatant was removed, and the amoebae were resuspended twice in sterile PAS. After the last centrifugation step, the amoebae were once again suspended in 30 ml of PAS supplemented with an antibiotic mix containing 10 μL of ciprofloxacin (4 μg/mL; Panpharma, Z.I., Clairay, France), 10 μL of vancomycin (4 μg/mL; Mylan, Saint- Priest, France), 10 μL of colimycin (500 IU/mL; Sanofi Aventis, Paris, France), 10 μL of rifampicin (4 μg/mL; Sanofi Aventis), and 10 μL of fungizone (100 μg/mL; Bristol-Myers Squibb, Rueil-Malmaison, France). The suspension was then dispensed in 0.5 ml amounts to the wells of a 24-well plate with a suspension cell concentration range of 1.10^6^–5.10^5^/ml. Each 100 μl sample was inoculated in the wells and incubated, taking into consideration amoebal growth temperature specificity in a humid chamber. These co-cultures were incubated for 4 days, and then sub-cultured twice on fresh amoebae in a one-tenth dilution as described above. After 3 days, the wells were observed under optical microscopy on the third passage and wells with amoebal lysis were further analyzed as positive for giant viruses. A negative amoebal control without any inoculated samples was used in each microplate.

### Cytospin and Staining

A 100 μl volume of amoebal culture presenting lysis was re-inoculated in a 1 ml suspension of fresh amoebae supplemented with an antibiotic mixture into 12-well plates (one-tenth dilution), then inoculated onto the same co-cultures following the procedures described above. After approximately 16–18 h, Amoeba became rounded, so 100 μl of the previously inoculated suspension was processed in the cytospin and fixed with methanol. The virus factories and viral particles were observed after hemacolor staining or fluorescence labeling (Hemacolor^®^, Merck, Darmstadt, Germany; Boughalmi et al., 2013a,b,c; Supplementary Figure S1).

### DNA Extraction and PCR Assays

In conjunction with the cytospin procedures, 200 μl of each inoculated suspension were used for DNA extraction. The remaining volume of the sample was frozen at -80°C for further use. Viral DNA was extracted with the automated EZ1 Virus Mini-Kit v.2 kit (Qiagen GmbH, Hilden, Germany) according to the manufacturer’s instructions. DNA quality and concentration were checked, using a nanodrop spectrophotometer (Thermo Scientific, Waltham, MA, USA). Real-time PCR to identify Marseilleviruses and Mimiviruses lineages based on hydrolysis probes was performed as described by Ngounga et al. (2013). In brief, tests were performed using the QuantiTec Probe PCR kit (Qiagen). PCR assays were performed using 5 μl of extracted DNA (∼50 nanograms) in an amplification reaction mix containing 12.5 μl of 2X QuantiTec Probe PCR Master Mix, 0.5 μl of probe at 1 pmol/μl, and 0.5 μl (0.2 μM) of forward and reverse primers for assays for lineage B Mimiviruses and Marseilleviruses or 1 μl (0.4 μM) of forward and reverse primers for assays for lineage A and C Mimiviruses. PCR assays were adjusted to a final volume of 25 μl by adding RNAse/DNAse-free water. PCRs were performed on a CFX96TM real-time system instrument (Bio-Rad, Hercules, CA, USA). The PCR amplification protocol was as follows: 15 min at 95°C followed by 45 cycles of denaturation at 95°C for 30 s and annealing/extension at 60°C for 1 min. A known lineage A, B, and C Mimivirus, and a Marseillevirus were used as positive controls.

Given the high genetic diversity of Mimiviruses and in case of negative results in real-time PCR, a standard PCR intended to be less susceptible to polymorphism was performed using primers targeting the mimivirus polymerase B gene (DNApol_R322 – forward 5′AAACAGGTGCACCAACATCA and reverse 5′GGTTTCCATTTTGACCCAAG). Assays were performed using the HotStarTaq DNA Polymerase kit (Qiagen). The PCR reactions were performed using 3 μl of extracted DNA (∼50 nanograms) in an amplification reaction mix containing 0.25 μl of Hot Start Taq polymerase (5 units/μl), 2.5 μl of buffer 10X, 2.5 μl of MgCl_2_ (25 mM), 2.5 μl of dNTPs and 1.0 μl (10 μM) of forward and reverse primers for each assay. Reactions were adjusted to a final volume of 25 μl by adding RNAse/DNAse-free water. The PCR amplification protocol was as follows: 15 min at 95°C followed by 40 cycles of denaturation at 95°C for 1 min, annealing at 56°C for 1 min and extension at 72°C. Positive DNA controls were lineage A, B, and C mimivirus strains routinely maintained in the laboratory. Afterwards, these samples were purified using NucleoFast plates (Macherey-Nagel GmbH & Co. KG, Duren, Germany), then sequenced with PCR primers used in the standard PCR assay with an ABI PRISM BigDye Terminator v3.1 Cycle Sequencing Kit (Applied Biosystems, Foster City, CA, USA) according to the manufacturer’s instructions. The sequences were assembled, analyzed using Chromaspro software (Technelysium) and compared with sequences in the GenBank database using BLAST software^1^

Viruses that were not detected by both real-time and standard PCRs were again tested with the hemacolor stain to detected optical structures identical to other viruses reported as described in section “Cytospin and Staining.”

### Fluorescent Labeling

In addition to hemacolor staining, used for preliminary morphological characterization, fluorescent labeling was used to visualize the viral factory. To do so, a 100 ml suspension of previously infected cells at a concentration of 4 × 10^5^ cells/ml was dispensed to a Cytospin chamber, centrifuged for 10 min at 800 rpm in a Shandon Cytospin 4 (Thermo Electron Corporation), then fixed for 10 min in methanol. For direct fluorescence with DAPI (49,69-diamidino-2-phenylindole) staining, cells were covered with 5 mM DAPI from a ready-to-use solution, “ProLong Gold Antifade Reagent” (Molecular Probes) and stained for 10 min in the dark prior to observation. The images were acquired with an LSM 510 Zeiss microscope, with DAPI staining observed using a UV diode (405 nm), *z* step = 0.3 mm (Suzan-Monti et al., 2007; Supplementary Figure S2).

### Electron Microscopy

Viruses not identified by PCR were also viewed by electron microscopy using the negative staining technique. In the grids after glow discharge, supernatant of the positive samples (20–40 μl) was added for 10 min to achieve adherence to the grids. The samples were washed, fixed and contrasted using a 5% solution of ammonium molybdate, again washed and dried before electron microscopic analysis. The samples were observed using a Morgagni 268 D (Philips) operating at 100 60 keV and a Tecnai G2 operating at 200 keV (Supplementary Figure S3).

**FIGURE 2:**
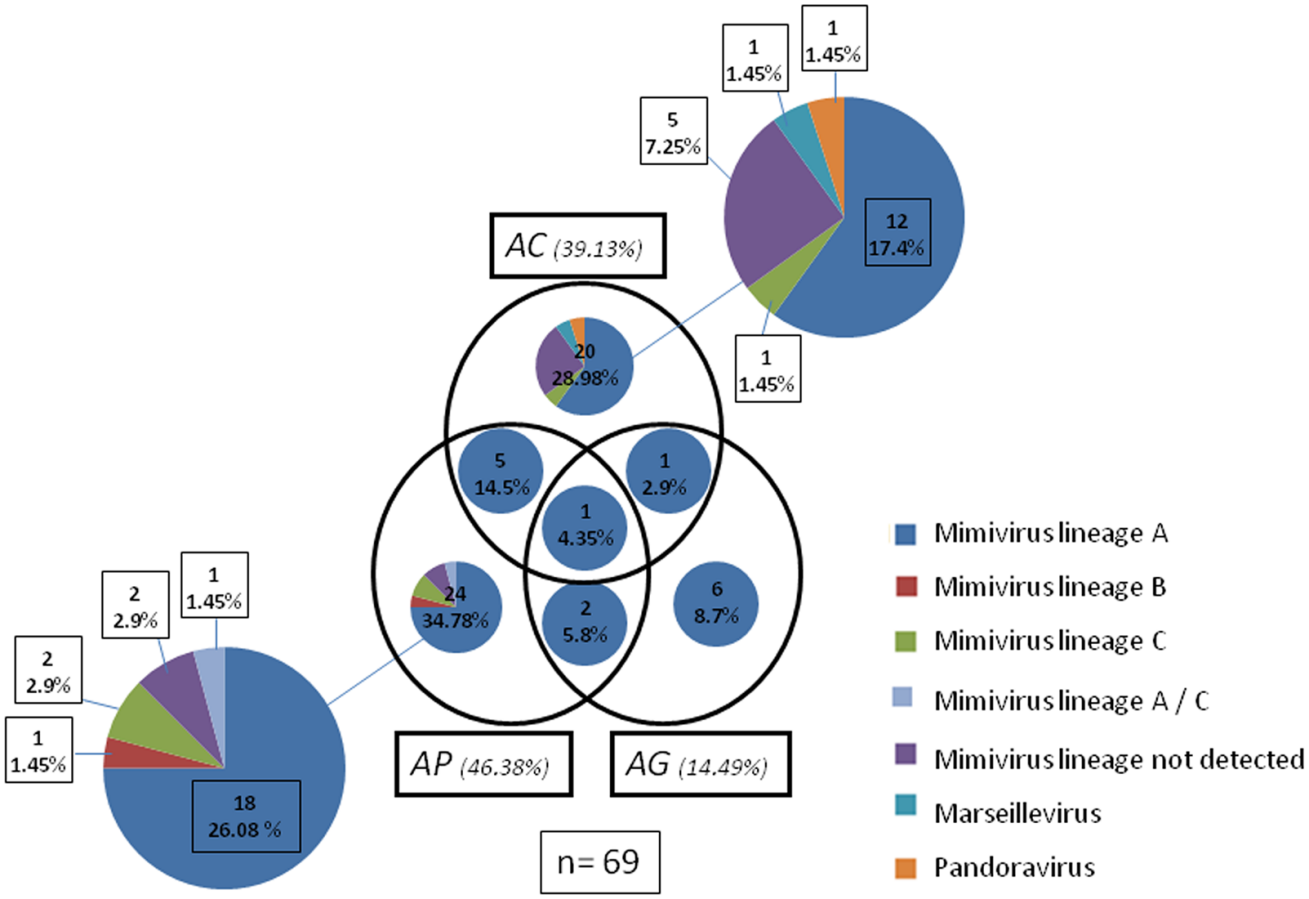
**Venn diagram, showing amoebas supports and isolated viruses.** A total of 69 virus were isolated in the three different platform cellular *AC Acanthamoeba polyphaga (AP)*, *Acanthamoeba griffinii (AG)* are represented. Viruses come from the same species and lineages detected by PCR are represented with the same color. The interception of the circle show viruses isolates coming from the same environmental samples in different *Acanthamoeba* platform of isolation. All the small graphics inside the circle represents the species and lineage detected by PCR. Mimivirus that were not detected by PCR is also represented. The number and the percentual of viruses isolated are also expressed.

The environmental giant virus samples are summarized in **Table 1** and results are expressed in **Figure 2**.

**Table 1:** Brazilian virus isolated in environmental samples represented in collection area, virus detected by PCR system, amoebas support and the positives methods.

	Sample	Amoeba support	Collection area	Identified virus(es)	Positive in the methods below
(1)	BZ 4	AP	1	Mimivirus A and B	qPCR, hemacolor
(2)	BZ 6^∗^	AP	1	Mimivirus A	qPCR
(3)	BZ 8	AP	1	Mimivirus A	PCR standard
(4)	BZ 16	AP	2	Mimivirus A	qPCR, electron microscopy
(5)	BZ 17	AP	2	Mimivirus A	qPCR
(6)	BZ 23	AP	2	Mimivirus C	qPCR
(7)	BZ 33	AP	2	Mimivirus A	PCR standard
(8)	BZ 34	AP	2	Mimivirus A	PCR standard
(9)	BZ 35^∗^	AP	2	Mimivirus A	PCR standard
(10)	BZ 37^∗^	AP	2	Mimivirus A	PCR standard
(11)	BZ 38^∗∗^	AP	2	Mimivirus A	PCR standard
(12)	BZ 39^∗^	AP	2	Mimivirus A	qPCR
(13)	BZ 40	AP	2	Mimivirus A	PCR standard
(14)	BZ 43	AP	2	Mimivirus A	PCR standard
(15)	BZ 46	AP	2	Mimivirus A	PCR standard
(16)	BZ 49	AP	2	Mimivirus B	qPCR
(17)	BZ 53	AP	2	Mimivirus A	PCR standard
(18)	BZ 71	AP	3	Mimivirus A	qPCR, DAPI, electron microscopy
(19)	BZ 72^∗^	AP	3	Mimivirus A	qPCR
(20)	BZ 76^∗^	AP	3	Mimivirus A	PCR standard
(21)	BZ 77	AP	3	Mimivirus A	PCR standard
(22)	BZ 82^∗^	AP	3	Mimivirus A	PCR standard
(23)	BZ 84	AP	3	Mimivirus A	PCR standard
(24)	BZ 87	AP	3	Mimivirus A	qPCR, hemacolor, DAPI, electron microscopy
(25)	BZ 88	AP	3	Mimivirus A	PCR standard, DAPI, electron microscopy
(26)	BZ 92	AP	3	Mimivirus A	qPCR
(27)	BZ 94	AP	3	Mimivirus C	qPCR
(28)	BZ 95	AP	3	Mimivirus A	PCR standard
(29)	BZ 96	AP	3	Mimivirus A	PCR standard
(30)	BZ 97	AP	3	Mimivirus A	PCR standard
(31)	BZ 98	AP	3	ND	Hemacolor
(32)	BZ 99	AP	3	ND	Hemacolor
(33)	BZ1	AC	1	Marseillevirus	qPCR, hemacolor, electron microscopy
(34)	BZ5	AC	1	Mimivirus A	PCR standard
(35)	BZ16	AC	2	Mimivirus C	qPCR, hemacolor
(36)	BZ20	AC	2	Mimivirus A	qPCR
(37)	BZ24	AC	2	ND	Hemacolor, electron microscopy
(38)	BZ28	AC	2	Mimivirus A with virophage	qPCR, electron microscopy
(39)	BZ31	AC	2	Mimivirus A	qPCR
(40)	BZ36	AC	2	Mimivirus A	PCR standard
(41)	BZ37^∗^	AC	2	Mimivirus A	qPCR
(42)	BZ38^∗∗^	AC	2	Mimivirus A	PCR standard
(43)	BZ39^∗^	AC	2	Mimivirus A	PCR standard
(44)	BZ41	AC	2	Mimivirus A	PCR standard
(45)	BZ58^∗^	AC	2	Mimivirus A	PCR standard
(46)	BZ72^∗^	AC	3	Mimivirus A	PCR standard
(47)	BZ74	AC	3	Mimivirus A	PCR standard
(48)	BZ75	AC	3	Mimivirus A	PCR standard
(49)	BZ76^∗^	AC	3	Mimivirus A	PCR standard
(50)	BZ79	AC	3	Mimivirus A	PCR standard
(51)	BZ81	AC	3	Pandoravirus	Hemacolor, PCR standart, electron microscopy
(52)	BZ82^∗^	AC	3	ND	Hemacolor
(53)	BZ83	AC	3	Mimivirus A	PCR standard
(54)	BZ86	AC	3	ND	Hemacolor
(55)	BZ92	AC	3	ND	Hemacolor
(56)	BZ95	AC	3	ND	Hemacolor
(57)	BZ98	AC	3	Mimivirus A	PCR standard
(58)	BZ99	AC	3	Mimivirus A	PCR standard
(59)	BZ100	AC	3	Mimivirus A	PCR standard
(60)	BZ6^∗^	AG	1	Mimivirus A	qPCR
(61)	BZ7	AG	1	Mimivirus A	qPCR
(62)	BZ32	AG	2	Mimivirus A	qPCR
(63)	BZ35^∗^	AG	2	Mimivirus A	qPCR
(64)	BZ38^∗∗^	AG	2	Mimivirus A	PCR standard
(65)	BZ45	AG	2	Mimivirus A	qPCR
(66)	BZ50	AG	2	Mimivirus A	qPCR
(67)	BZ56	AG	2	Mimivirus A	qPCR
(68)	BZ58^∗^	AG	3	Mimivirus A	qPCR
(69)	BZ68	AG	3	Mimivirus A	PCR standard

*^∗^Virus isolated in two amoebas support coming from the same environmental sample, ^∗∗^virus isolated in three amoebas support coming from the same environmental sample.*

### Polymorphism

The DNA polymerase B amplification difficulties may be due to the high genetic diversity of the viruses, resulting in low adherence of some giant virus primers in regions with high polymorphism. Optimal alignment of the predicted highly conserved DNA polymerase B amplicons sequences were analyzed using MEGA version 6.0^2^

## Results

### Isolation of Brazilian Giant Viruses

A total of 69 viruses were isolated in three different cellular supports (*n* = 69; **Table 1**). The highest isolation percentages were in AP (46.38%) followed by AC (39.13%) and AG (14.49%). No virus was isolated in VV. Among all the viruses isolated, the most common was lineage A Mimivirus (79.73%), followed by lineage C Mimivirus (4.35%), and lineage B Mimivirus (1.45%). Undetected Mimivirus lineages represented 10.15% of isolates. One Marseillevirus (1.45%) and one Pandoravirus (1.45%) were also isolated. In AG Mimivirus, only lineage A was isolated, while AP allowed isolation of all lineages of Mimiviruses and AC demonstrated possible isolation of the different giant virus species Marseillevirus and Pandoravirus. (**Table 1**; **Figure 2**).

Of all viruses coming from the same environmental sample, one lineage A mimivirus was detected in each of the three *Acanthamoeba* cell supports (4.35%). In addition, AP and AC gave five lineage A Mimiviruses from the same environmental sample (14.5%), while AP and AG gave two mimiviruses (5.8%) and AC and AG, two viruses (2.9%). (**Table 1**; **Figure 2**).

Viruses not detected by the PCR system (10.15%) were viewed by hemacolor staining. Mimivirus-like structures and viral factories inside the cytoplasm were detected in slide stains (**Table 1**; **Figure 2**) (Supplementary Figure S1). Some viruses were also visualized with fluorescent labeling using the DAPI and could be visualized the viral factory (Supplementary Figure S2). Negative staining was also performed for some giant viruses (Supplementary Figure S3).

In the correlation between the three collection areas, six giant viruses (8.7%) were isolated from Area 1, 31 (44.93%) from Area 2 and 32 (46.38%) from Area 3 (**Figure 1**; **Table 1**). Comparing cellular supports, three giant viruses (4.35%) from Area 1, 14 giant viruses from Area 2 (20.29%) and 15 giant viruses from Area 3 (21.74%) were isolated in AP. One giant virus (1.45%) from Area 1, 11 giant viruses from Area 2(15.94%) and 15 from Area 3 (21.74%) were isolated in AC. Two giant viruses (2.9%) from Area 1, six viruses from Area 2 (8.7%), and two viruses from Area 3 (2.9%) were isolated in AG.

### Polymorphism

**FIGURE 3:**
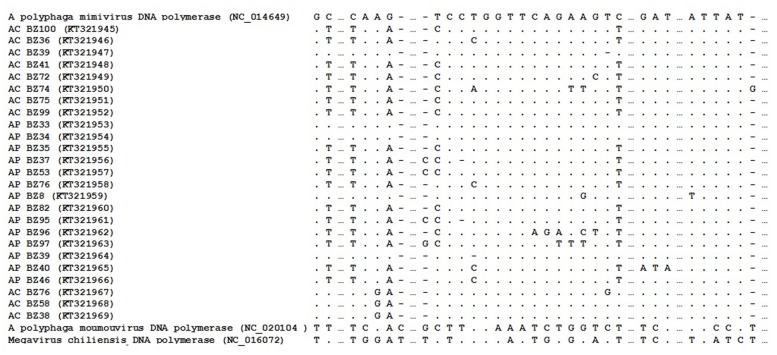
**Nucleotides sequence of a fragment of the mimivirus DNA polymerase B gene.** Samples obtained in this study are underlined; boldface indicates polymorphic.

Optimal alignment of the predicted highly conserved DNA polymerase B gene sequences showed several polymorphism substitutions in the mimivirus amplicons we derived compared to other available sequences (**Figure 3**). The sequences have been deposited in GenBank under the following accession numbers: KT321945-KT321969.

## Discussion

Using inoculation of a panel of protozoa as supports for co-culture, we were able to isolate 69 giant viruses from Brazilian environmental samples. Since the first reported mimivirus isolation (La Scola et al., 2003), many giant viruses have been detected and isolated from diverse environments and even in humans (La Scola et al., 2003, 2008; Arslan et al., 2011; Saadi et al., 2013a,b; Campos et al., 2014; Dornas et al., 2014a; Scheid et al., 2014; Andrade et al., 2015; Assis et al., 2015; Reteno et al., 2015). Isolation and detection have occurred at different rates in the different samples studied, with low positive detection and isolation in bronchoalveolar lavages and feces (Saadi et al., 2013a,b) and more strongly positive results in soil, water, and sewage (Pagnier et al., 2013; Campos et al., 2014; Dornas et al., 2014a,b; Assis et al., 2015; Reteno et al., 2015). The environmental samples chosen herein were previously described as sources for isolation of numerous giant viruses (Gaze et al., 2011; Pagnier et al., 2013; Philippe et al., 2013; Assis et al., 2015; Reteno et al., 2015) and represent a good set of samples to compare different amoeba in their ability to isolate giant viruses.

Due to the obstacle and delay of giant virus isolation, the conventional isolation method was modified. The sample concentration by filtration was first reported by La Scola et al. (2008). The technique was subsequently supplemented with antibiotics in the co-culture to reduce bacterial overgrowth (La Scola et al., 2010). The high concentration of antibiotics, however, might have affected amoeba growth and consequently decreased the number of new giant viruses isolated. The procedure was tentatively improved using pre-enrichment, consisting of incubating the sample in a dark chamber with organic sources, thus allowing the multiplication of heterotrophic bacteria, and later inoculating the monolayer of amoebae (Arslan et al., 2011). The isolation method was modified adding free AC amoeba in a half-water/half-rice medium for about 30 days (Dornas et al., 2014b).

With this modified method it was possible to isolate some giant viruses of lineage A in Brazilian ecosystems (Campos et al., 2014; Andrade et al., 2015; Assis et al., 2015). Although this method is capable of isolating lineage A Mimiviruses, the previous one demonstrated low rate of positivity (1.2%), suggesting that the direct inoculation chosen for this project is more sensitive than previously reported methods (Arslan et al., 2011; Dornas et al., 2014b).

In this vein, seeking to increase the isolation of giant viruses, a high-throughput method, previously developed for isolation of Phycodnaviruses (Fitzgerald et al., 2010) in algae cells, was standardized for species-dependent isolation of the *Acanthamoeba* giant virus sp. With this method, over 1000 samples were tested, resulting in the isolation of several giant viruses, showing that this method could be quickly used for large collections of environmental samples (Boughalmi et al., 2013a).

With these increased isolation possibilities, new samples began to be researched in the invertebrate group, such as larvae from the *Hirudo medicinalis* species (Boughalmi et al., 2013b) and leeches from the *Eristalis tenax* (Boughalmi et al., 2013c). The species were first disinfected with alcohol (Slimani et al., 2013), then the organ parts were macerated separately and subsequently inoculated in amoebal co-culture on agar plates (Boughalmi et al., 2013a).

Though all isolation methods have been presented as an evolution in isolation techniques, however, there remains a large discrepancy between the frequency of detection by molecular methods and the actual frequency of isolation (Ghedin and Claverie, 2005; Kristensen et al., 2010). One possible explanation for this is the great diversity of potentially existing giant viruses, which theoretically require a wide range of amoebal cells as supports for co-culture.

As mentioned, with a low positivity rate for the isolation method, molecular biology techniques for detection of giant viruses have evolved independently. Concerning the number of isolates whose genomes were sequenced, however, the only increase reported was in generic primers to the mimivirus lineage or other species such as *Marseillevirus* sp., *Pandoravirus* sp., and *Faustovirus* sp. (La Scola et al., 2010; Boughalmi et al., 2013a; Ngounga et al., 2013; Pagnier et al., 2013; Philippe et al., 2013). High genetic diversity may, however, explain the difficulty in amplifying some preserved lineage-specific regions, as described by genetic polymorphisms (Dornas et al., 2014a; Andrade et al., 2015; Santos Silva et al., 2015). Notably, the reason for PCR-negative giant viruses previously detected by lysis of amoebae and viewed by optical microscopy in stains was carefully analyzed in this work.

Although isolation of giant viruses has been reported using AC or VV as cell supports, most were isolated using AP, as also represented in this work (La Scola et al., 2008, 2010; Arslan et al., 2011; Boughalmi et al., 2013a; Pagnier et al., 2013; Saadi et al., 2013a; Scheid et al., 2014; Reteno et al., 2015). However, the AC amoeba has already been demonstrated to be effective in the isolation of giant viruses, especially in the isolation of a higher diversity of virus families such as Pandoravirus and Pithovirus, whereas AP appears to be more specific to Mimiviruses. In the present study, AG was used for the first time as an amoebal support for isolation of giant viruses. It seems to possess less isolation sensitivity and specificity than other cell supports, though it is still impossible to conclude whether this is due to this particular amoeba or to the incubation temperature used for this species, which could have limited primo-isolation of giant viruses.

Although, of all viruses isolated from Brazilian samples, there is a high rate of Mimiviruses lineage A (Campos et al., 2014; Andrade et al., 2015; Assis et al., 2015), as reported in this study, other mimivirus lineage B or C have also been reported, along with Marseillevirus and Pandoravirus. Surprisingly by comparing the different amoebal species for cellular supports, few giant viruses were isolated from the same environmental sample (**Table 1**). This suggests a tropism involving a close relationship between virus and host. Possible explanations could be genetic diversity, such as genes involved in RNA translation, the gene translation process and also factors involved in viral multiplication. Moreover, this tropism may suggest that giant viruses evolved together with their host.

The high positivity in Area 2, after chemical water treatment as well in Area 3, an isolated point of the lagoon, suggests that chemical treatment alone may not be sufficient to eliminate Mimivirus from water. Biocides are not totally effective in eliminating Mimivirus, varying in treatment time and composition (Campos et al., 2012). Also, the virus can be eliminated by chemical treatment and by contact with environmental soil; the virus multiplies better at in the treatment station than in sewage, where we have wider competition with many bacteria and other viruses. The low positivity in the sewage sample suggests that the virus may not multiply better than in water or even soil, suggesting that high microorganism concentration generates competition and this competition may interfere with multiplication. The Marseillevirus was isolated upstream of chemical sewage treatment and the Pandoravirus from an isolated area of the lagoon. For the rare giant viruses isolated from sewage, it will be difficult to correlate with the isolates from sewage treatment. In other terms, the fact that we isolated only one Marseillevirus from sewage can’t permit us to correlate between chemically treated sewage and sewage distant areas, because the diversity is of a big importance to have correlation between environments. We can speculate an hypothesis in this work to try understanding how these viruses circulate in nature. Through these results, we can suggest that some species of giant viruses might display specificity for an *Acanthamoeba* host. Also, with Brazilian environmental samples, we confirmed the high rate of Mimivirus lineage A, as well as differences in viruses affinity for different amoeba used as platforms for the isolation procedure.

## Conflict of Interest Statement

The authors declare that the research was conducted in the absence of any commercial or financial relationships that could be construed as a potential conflict of interest.

## Footnotes

1 http://blast.ncbi.nlm.nih.gov/

2 www.megasoftware.net

## Supplementary material

FIGURE S1 **Images obtained from the hemacolor staining of some viruses isolated.** Cytospin, fixation and stain were done after 18 h post infection. The asterisks in the image show the viral factory and the arrows in **Figure 2**. **(G)** show the Pandora’s virus particle. Original magnification ×1000. **(A)** Negative control *Acanthamoeba castellanii*; **(B)** AC BZ 01 Marseillevirus; **(C)** AC BZ 16 Mimivirus; **(D)** AC BZ 24 Mimivirus; **(E)** AP BZ 04 Mimivirus; **(F)** AP BZ 87 Mimivirus; **(G)** AC BZ 81 Pandoravirus.

FIGURE S2 **Images obtained from the labeling fluorescence DAPI of some viruses isolated and inoculated in same cellular system isolated.** Cytospin, fixation and labeling fluorescence were done after 18 h post infection. Viral factory were showed in the images. Original magnification ×1000. **(A)** Negative control *A. polyphaga*; **(B)** AP BZ 88; **(C)** AP BZ 87; **(D)** AP BZ 71.

FIGURE S3 **Negative staining of the Brazilian giant viruses isolate.** Supernatant of the sample were used18 h post infection. **(A)** AC BZ 01 Marseillevirus; **(B)** AC BZ 28 Mimivirus; **(C)** Virophage particles found in AC BZ 28; **(D)** AP BZ 16 Mimivirus; **(E)** AC BZ 81 Pandoravirus; **(F)** AP BZ 87 Mimivirus.
