# Supplementary figures and images for: Isolation of new Brazilian giant viruses from environmental samples using a panel of protozoa
Source: Front Microbiol. 2015 Oct 6;6:1086. doi: 10.3389/fmicb.2015.01086 (PMC4594340; doi:10.3389/fmicb.2015.01086)

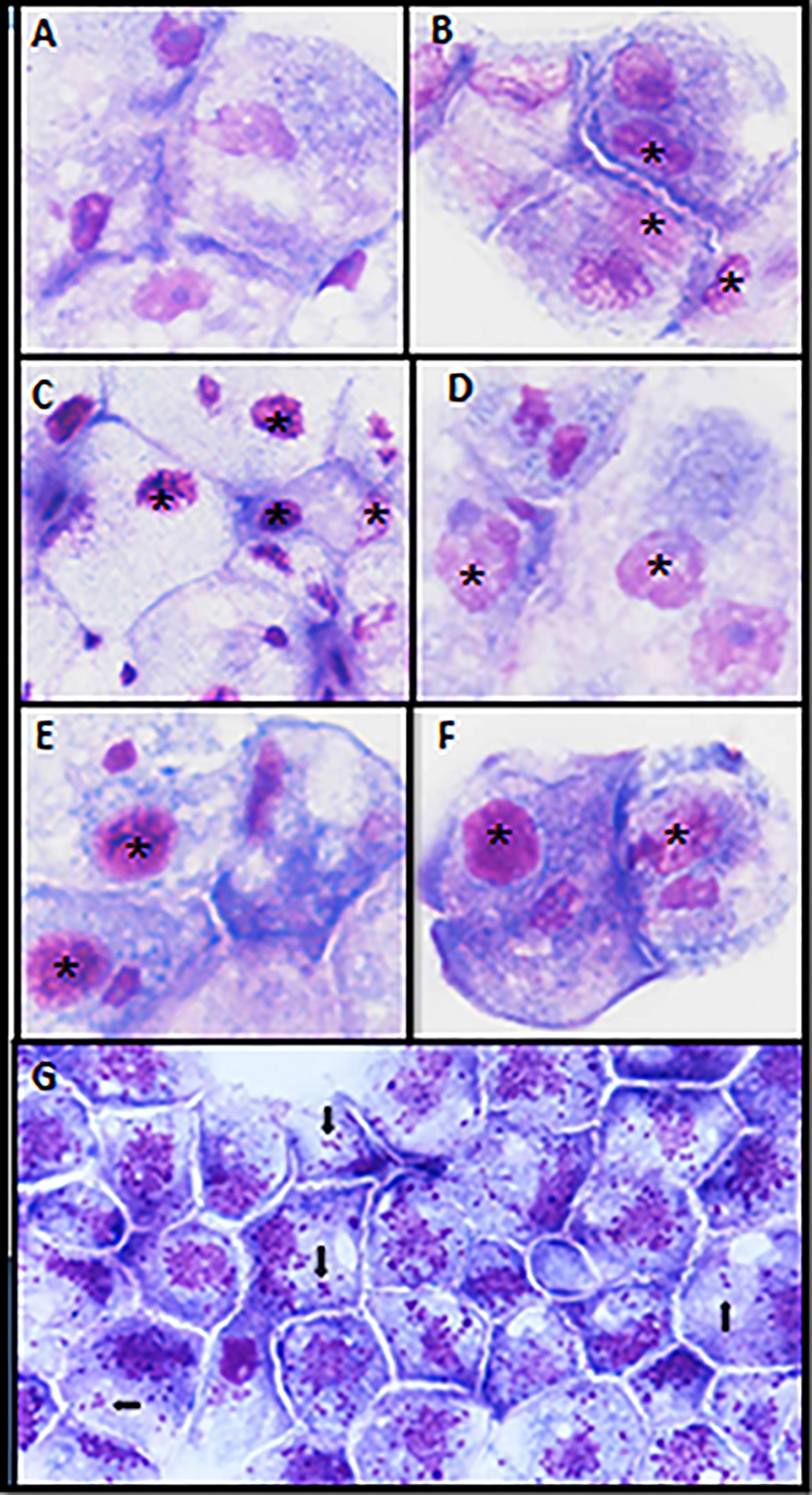

Supplement: FIGURE S1 — Images obtained from the hemacolor staining of some viruses isolated. Cytospin, fixation and stain were done after 18 h post infection. The asterisks in the image show the viral factory and the arrows in Figure 2. (G) show the Pandora’s virus particle. Original magnification ×1000. (A) Negative control Acanthamoeba castellanii; (B) AC BZ 01 Marseillevirus; (C) AC BZ 16 Mimivirus; (D) AC BZ 24 Mimivirus; (E) AP BZ 04 Mimivirus; (F) AP BZ 87 Mimivirus; (G) AC BZ 81 Pandoravirus. [file Image_1.TIF]

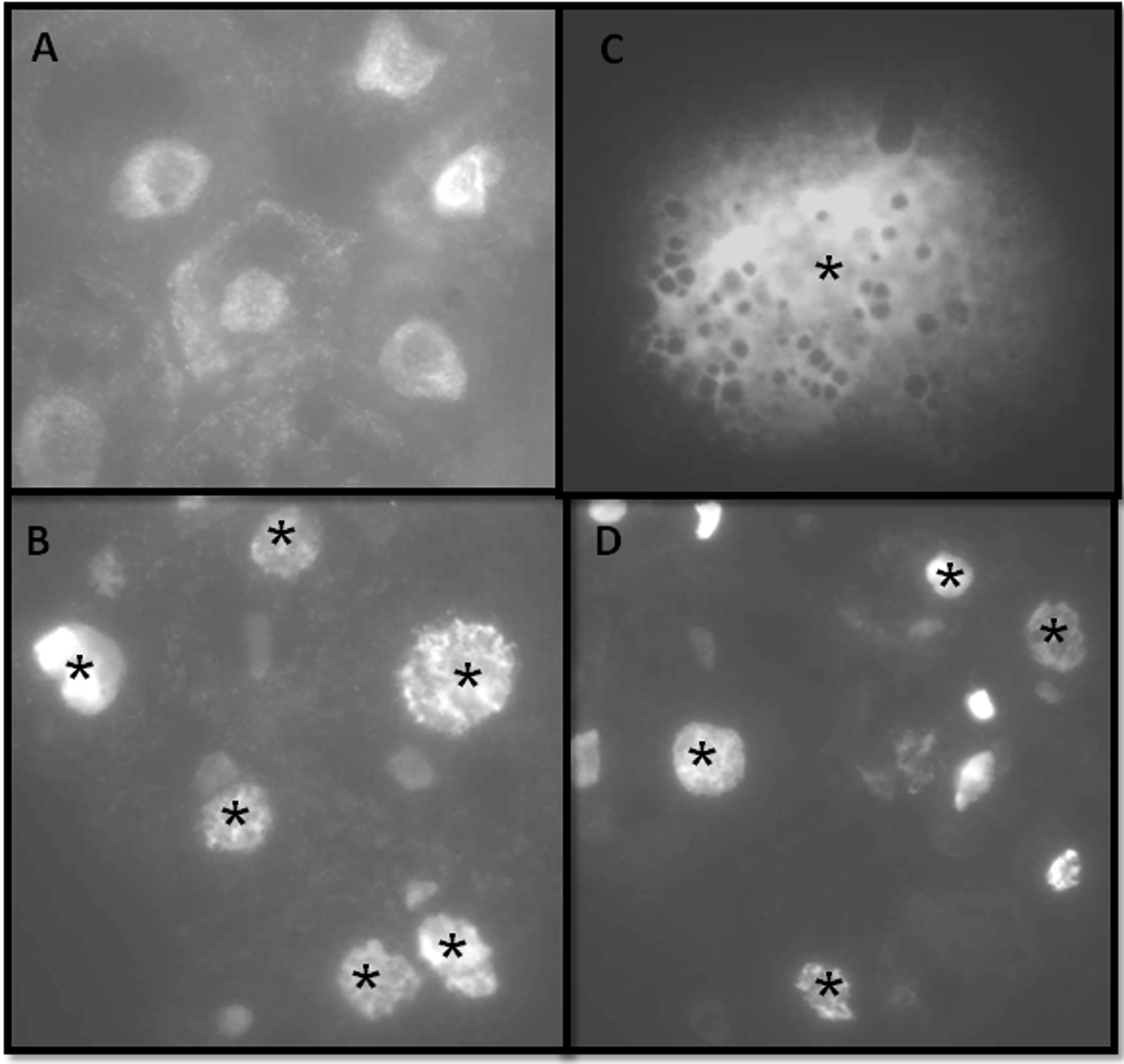

Supplement: FIGURE S2 — Images obtained from the labeling fluorescence DAPI of some viruses isolated and inoculated in same cellular system isolated. Cytospin, fixation and labeling fluorescence were done after 18 h post infection. Viral factory were showed in the images. Original magnification ×1000. (A) Negative control A. polyphaga; (B) AP BZ 88; (C) AP BZ 87; (D) AP BZ 71. [file Image_2.TIF]

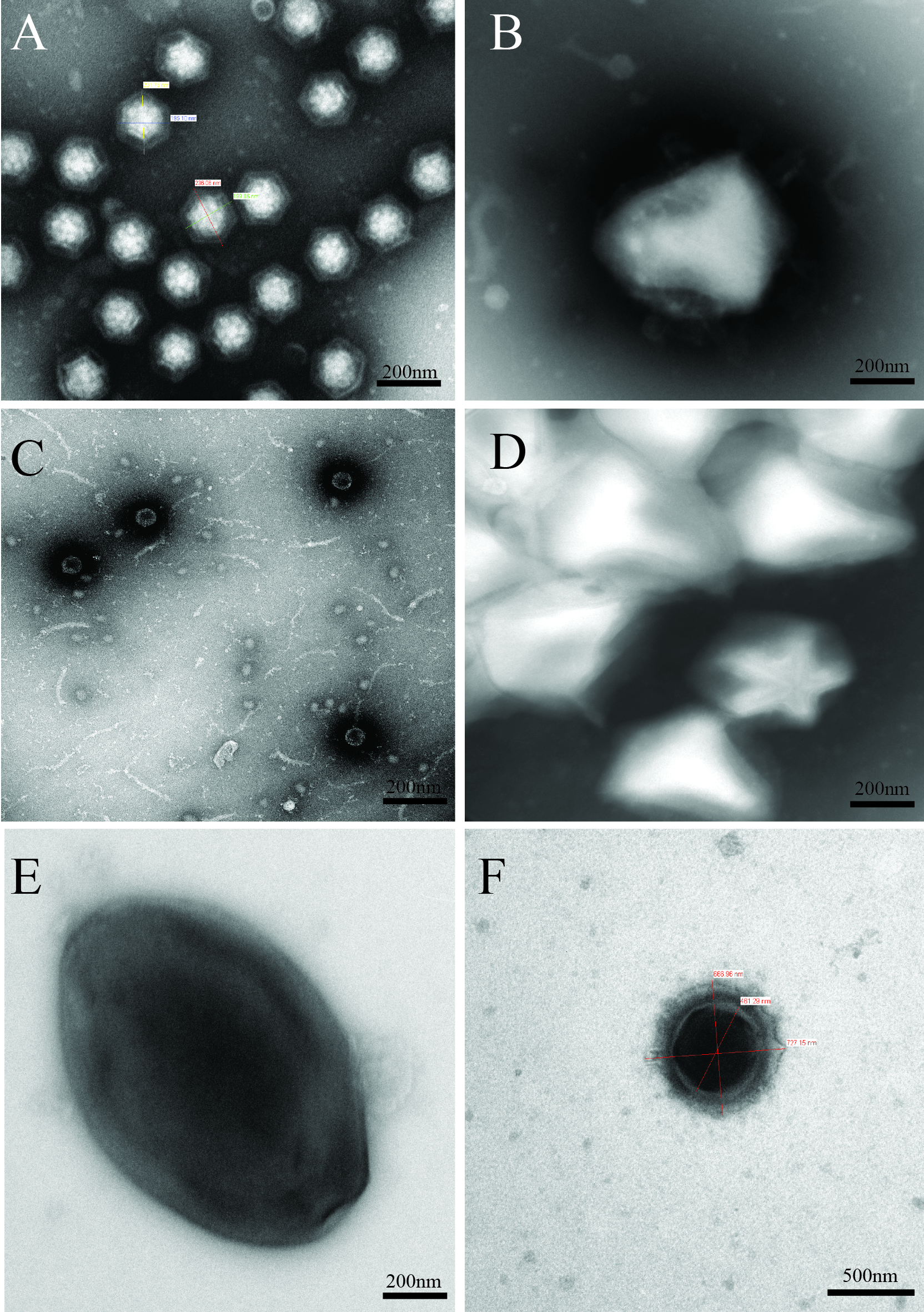

Supplement: FIGURE S3 — Negative staining of the Brazilian giant viruses isolate. Supernatant of the sample were used18 h post infection. (A) AC BZ 01 Marseillevirus; (B) AC BZ 28 Mimivirus; (C) Virophage particles found in AC BZ 28; (D) AP BZ 16 Mimivirus; (E) AC BZ 81 Pandoravirus; (F) AP BZ 87 Mimivirus. [file Image_3.TIF]
